# A short amphipathic alpha helix in scavenger receptor BI facilitates bidirectional HDL-cholesterol transport

**DOI:** 10.1016/j.jbc.2022.102333

**Published:** 2022-08-01

**Authors:** Sarah C. May, Daisy Sahoo

**Affiliations:** 1 Department of Medicine, Division of Endocrinology & Molecular Medicine, Medical College of Wisconsin, Milwaukee, Wisconsin, USA; 2 Department of Biochemistry, Medical College of Wisconsin, Milwaukee, Wisconsin, USA; 3 Cardiovascular Center, Medical College of Wisconsin, Milwaukee, Wisconsin, USA

**Keywords:** cholesterol, scavenger receptor, high-density lipoprotein, receptor structure-function, cardiovascular disease, mutant, homology modeling, protein structure, structural biology, atherosclerosis, GAPDH, glyceraldehyde 3-phosphate dehydrogenase, LIMP-2, lysosomal integral membrane protein 2, MUSCLE, MUltiple Sequence Comparison by Log Expectation, SD, standard deviation, SR-BI, scavenger receptor class B type I, WT, wildtype

## Abstract

During reverse cholesterol transport, high-density lipoprotein (HDL) carries excess cholesterol from peripheral cells to the liver for excretion in bile. The first and last steps of this pathway involve the HDL receptor, scavenger receptor BI (SR-BI). While the mechanism of SR-BI-mediated cholesterol transport has not yet been established, it has long been suspected that cholesterol traverses through a hydrophobic tunnel in SR-BI’s extracellular domain. Confirmation of a hydrophobic tunnel is hindered by the lack of a full-length SR-BI structure. Part of SR-BI’s structure has been resolved, encompassing residues 405 to 475, which includes the C-terminal transmembrane domain and its adjacent extracellular region. Within the extracellular segment is an amphipathic helix (residues 427–436, referred to as AH(427–436)) that showed increased protection from solvent in NMR-based studies. Homology models predict that hydrophobic residues in AH(427–436) line a core cavity in SR-BI’s extracellular region that may facilitate cholesterol transport. Therefore, we hypothesized that hydrophobic residues in AH(427–436) are required for HDL cholesterol transport. Here, we tested this hypothesis by mutating individual residues along AH(427–436) to a charged residue (aspartic acid), transiently transfecting COS-7 cells with plasmids encoding wild-type and mutant SR-BI, and performing functional analyses. We found that mutating hydrophobic, but not hydrophilic, residues in AH(427–436) impaired SR-BI bidirectional cholesterol transport. Mutating phenylalanine-430 was particularly detrimental to SR-BI’s functions, suggesting that this residue may facilitate important interactions for cholesterol delivery within the hydrophobic tunnel. Our results support the hypothesis that a hydrophobic tunnel within SR-BI mediates cholesterol transport.

Atherosclerosis, the most common type of cardiovascular disease, occurs when excess cholesterol accumulates in plaques along the artery wall. This plaque accumulation restricts blood flow through the arteries and may lead to heart attack or stroke. Cholesterol is carried by two main lipoprotein particles: low-density lipoprotein (LDL) and high-density lipoprotein (HDL). While LDL deposits cholesterol in arterial plaques, HDL removes excess cholesterol from circulation. This latter process, known as reverse cholesterol transport, requires the HDL receptor, scavenger receptor BI (SR-BI).

SR-BI is an 82-kDa cell surface protein with a large extracellular domain that is anchored by two transmembrane domains with short N- and C-terminal intracellular tails. SR-BI binds HDL and facilitates selective uptake of HDL cholesteryl esters (HDL-CEs) into the liver for subsequent excretion (1). SR-BI also effluxes free cholesterol from cells, including macrophages in the artery wall, to HDL particles (2). The precise mechanisms of SR-BI-mediated cholesterol transport between HDL and cells are not well understood, but one hypothesis is that cholesterol transport occurs through a predicted hydrophobic channel spanning the length of the extracellular domain (3, 4).

By X-ray crystallography, the exoplasmic domains of related class B scavenger receptors, CD36 and lysosomal integral membrane protein 2 (LIMP-2), were shown to possess hydrophobic tunnels containing fatty acids (5) or cholesterol (6), respectively. There is strong evidence that a hydrophobic cavity also exists within SR-BI’s extracellular domain. Namely, blocker of lipid transport-1 (BLT-1)’s ability to block SR-BI-mediated cholesterol transport depends on the presence of a single residue, cysteine-384 (7), which is predicted to reside within a hydrophobic cavity in SR-BI homology models (3). As the full-length structure of SR-BI has not yet been solved, we are currently unable to verify that the predicted hydrophobic cavity exists. We previously solved a partial NMR structure of an SR-BI peptide (residues 405–475), which includes the C-terminal transmembrane domain and nearby extracellular domain. A subset of these extracellular residues (427–436) comprises an amphipathic helix (“AH”; originally referred to as “Helix 2”) (8), hereafter referred to as AH(427–436). Because AH(427–436) exists in SR-BI’s extracellular domain, it is anticipated that the hydrophobic residues in AH(427–436) are embedded in the core of this domain (*i.e.*, where cholesterol transport between HDL and the membrane is thought to occur). Further, because of its proximity to the transmembrane domains, AH(427–436) may reside at an entry point of cholesterol into plasma membranes, suggesting that hydrophobic residues in AH(427–436) may line the hydrophobic tunnel. Considering this, we hypothesized that hydrophobic residues in AH(427–436) are required for facilitating SR-BI-mediated cholesterol transport.

To test our hypothesis, we generated two sets of SR-BI mutant receptors. We disrupted the hydrophobicity of AH(427–436) by substituting a charged residue, aspartic acid (D), for individual residues along the hydrophobic face of AH(427–436) in full-length murine SR-BI. Single-point mutations were made at the following residues: leucine-427 (L427), phenylalanine-430 (F430), tyrosine-431 (Y431), leucine-434 (L434), and valine-435 (V435). Additionally, we made substitutions along the hydrophilic face of AH(427–436) at the following residues: serine-428 (S428), threonine-429 (T429), threonine-432 (T432), glutamine-433 (Q433), and leucine-436 (L436). Following transient transfection of COS-7 cells with plasmid encoding empty vector, murine wildtype (WT) SR-BI, or mutant SR-BI constructs, HDL cholesterol transport functions were assessed.

## Results

### The near C-terminal extracellular region of SR-BI contains a conserved, amphipathic alpha helix

**Figure 1:**
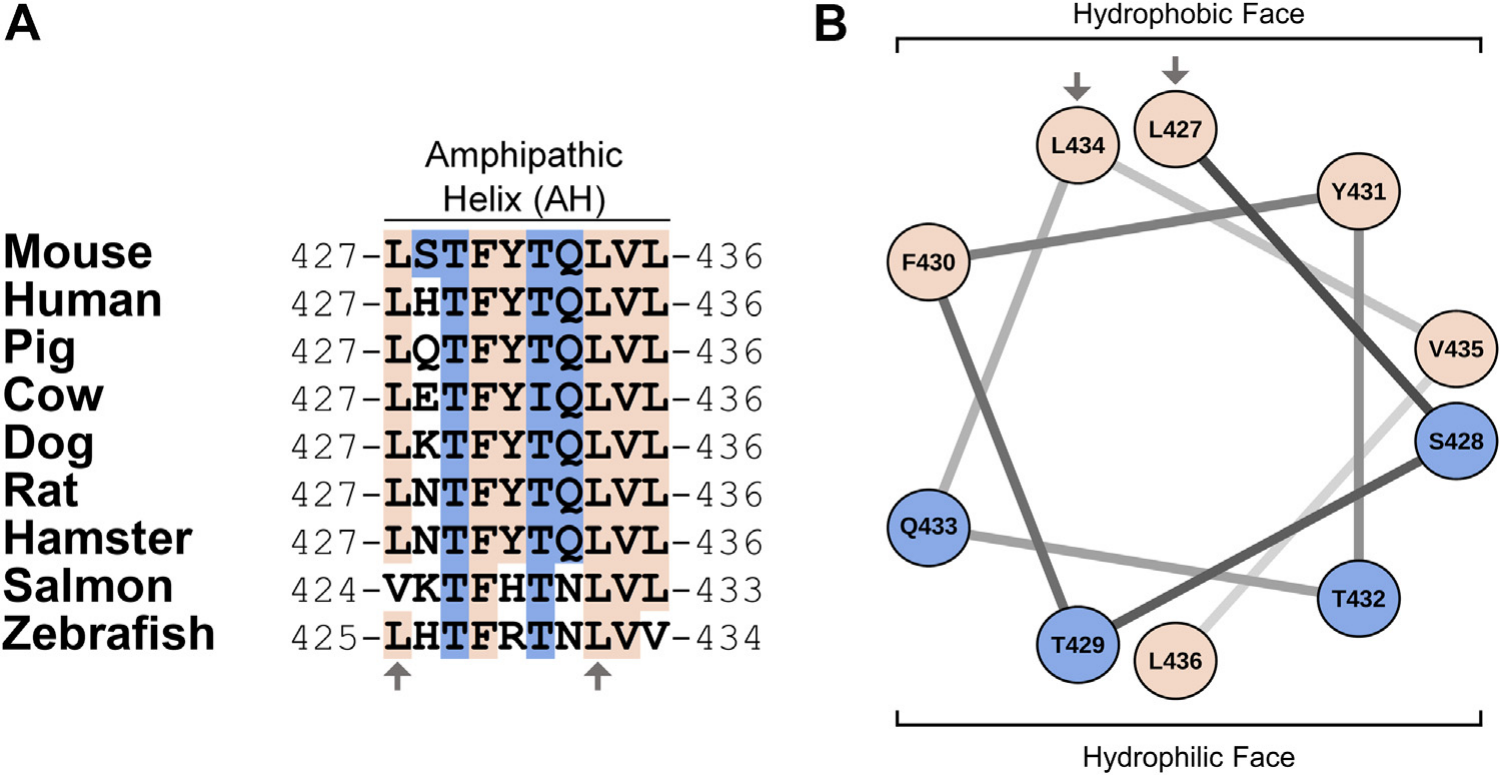
**The near C-terminal extracellular region of SR-BI contains a conserved, amphipathic alpha-helix.***A*, the amino acid sequence of murine SR-BI was aligned with other species using the MUSCLE algorithm (9). Hydrophobic and hydrophilic residues that are fully conserved between mouse and other species are colored in *tan* and *blue*, respectively. Residues that are within the leucine zipper dimerization motif are indicated by *arrows*. *B*, a helical wheel diagram for AH(427–436) (residues 427–436) was generated with the NetWheels server, using the same coloration and symbols as in (*A*). SR-BI, scavenger receptor class B type I.

The full-length amino acid sequences of SR-BI across various species were aligned using the MUSCLE algorithm (9). The sequence alignment for the region of AH(427–436) is shown (Fig. 1*A*). Conserved residues were colored according to whether they are hydrophobic (tan) or hydrophilic (blue). T429, F430, T432, L434, and V435 were fully conserved in terms of sequence identity. L427, Q433, and L436 were substituted with amino acids having similar biochemical properties in salmon and/or zebrafish. Conversely, hydrophobic Y431 was substituted with a positively charged amino acid in salmon and zebrafish. The identity of residue 428 was more variable, but most substitutions were either hydrophilic and uncharged (*e.g.*, S428 in mouse) or hydrophilic and basic (*e.g.*, histidine-428 in human). The exception was bovine SR-BI, which has a negatively charged glutamic acid at this position. Overall, the amphipathicity of AH(427–436) is highly conserved, particularly in higher vertebrates. To highlight this amphipathicity, we generated a helical wheel diagram for AH(427–436) (Fig. 1*B*) using the NetWheels server (http://lbqp.unb.br/NetWheels/). This shows that hydrophobic residues cluster along one face of AH(427–436), while the other face is primarily hydrophilic. AH(427–436) encompasses two leucine residues (L427 and L434) of a previously reported leucine zipper dimerization motif (8), as indicated by arrows.

### SR-BI’s AH(427–436) may support a hydrophobic tunnel that facilitates cholesterol transport

**Figure 2:**
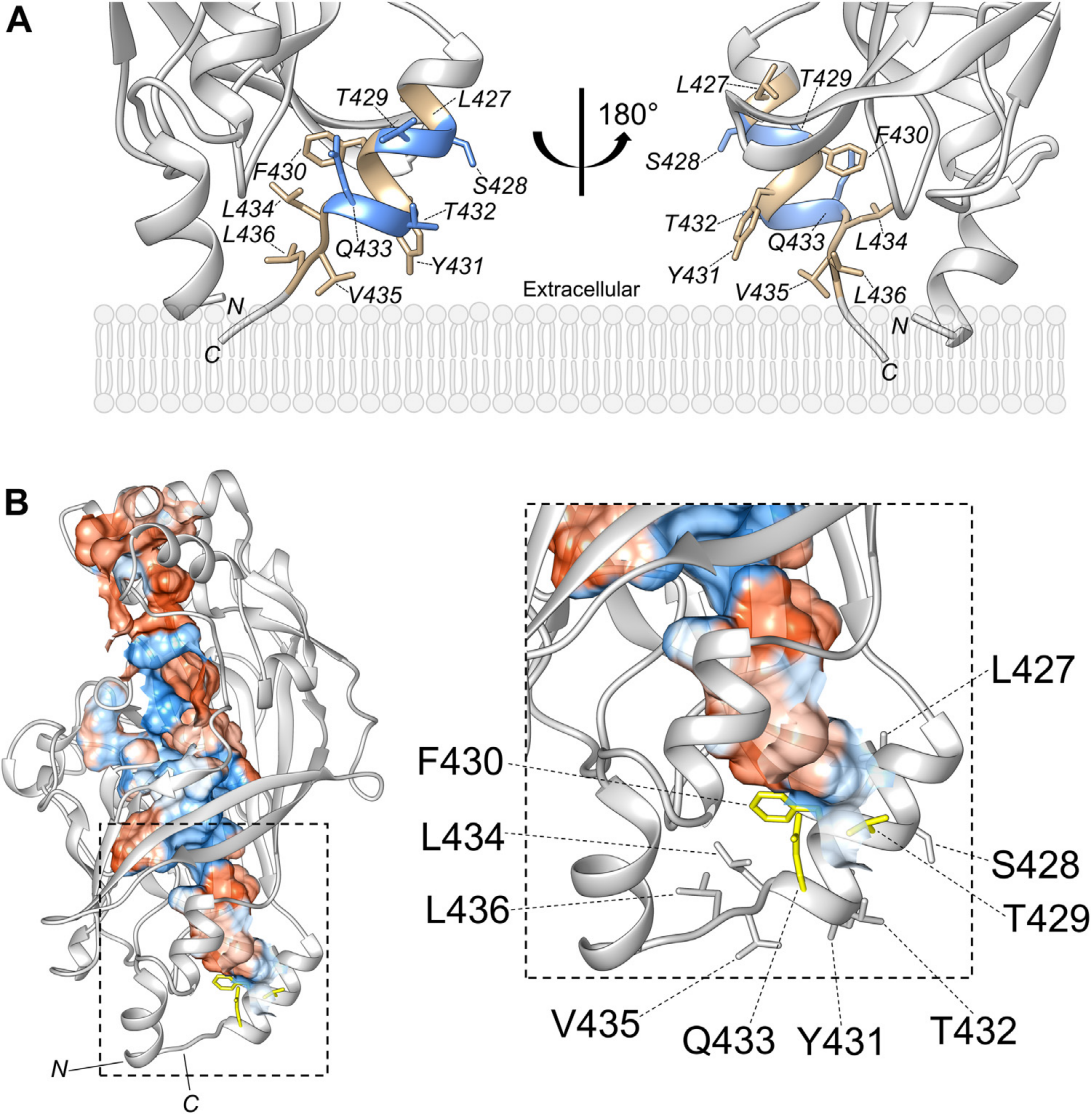
**SR-BI’s AH(427–436) may support a hydrophobic tunnel that facilitates cholesterol transport**. *A*, an extracellular homology model of murine SR-BI was generated using MODELLER and UCSF Chimera. A zoomed-in view of AH(427–436) at the base of the extracellular domain is shown with hydrophilic residues colored in *blue* and hydrophobic residues colored in *tan* and rotated 180° around its vertical axis. Plasma membrane bilayer is included as a general guide and is not to scale. *B*, CASTp was used to identify cavities within the SR-BI homology model, and the largest solvent-accessible cavity is colored according to hydrophobicity (*red* = hydrophobic, *blue* = hydrophilic). An enlarged view of the area within the dashed box shows the orientations of AH(427–436) residues in relation to the cavities. Residues in *yellow* may be part of the hydrophobic cavity. SR-BI, scavenger receptor class B type I.

One hypothesized mechanism of SR-BI-mediated HDL cholesterol transport is through a hydrophobic tunnel that spans the extracellular domain (4). SR-BI homology models predict the presence of a hydrophobic tunnel that is large enough to transport free cholesterol and CE (3). These models suggest that a hydrophobic tunnel would shuttle cholesterol from SR-BI’s extracellular domain to the area of the plasma membrane immediately surrounding SR-BI’s transmembrane domains, a possible point of entry into the cells. Because AH(427–436) is near the C-terminal transmembrane domain, we suspected that AH(427–436) may be an integral component of the predicted hydrophobic tunnel. First, we generated a homology model of murine SR-BI using MODELLER (10) with X-ray crystal structures of LIMP-2 (PDB ID: 4F7B) and CD36 (PDB ID: 5LGD) used as templates. AH(427–436) residues were highlighted using UCSF Chimera software (11, 12). There is strong overlap in this region between murine and human SR-BI (data not shown). As shown in the homology model (Fig. 2*A*), residues along the hydrophobic face of AH(427–436) extend into the core of the extracellular domain, while hydrophilic residues along SR-BI-AH extend into the extracellular space. Notably, L434, V435, and L436 appear to be in an unstructured region (Fig. 2*A*). Because these residues are near the end of the domains for which LIMP-2 and CD36 X-ray crystal structures were solved, we cannot rule out potential artifacts created by structural determination conditions. Other homology models of the entire SR-BI molecule include L434, V435, and L436 within the amphipathic alpha helix (13). Using the Computed Atlas of Surface Topography of proteins (CASTp) server (14), solvent-accessible pockets were located. The pocket with the largest area is shown, colored by hydrophobicity (hydrophobic = red, hydrophilic = blue), with residues contacting the cavity colored in yellow (Fig. 2*B*). One hydrophobic residue, F430, is at the base of this hydrophobic cavity (Fig. 2*B*). Two hydrophilic residues, T429 and Q433, are present at the opening of this cavity.

### Effects of aspartic acid mutations on mean hydrophobicity and amphipathicity of AH(427–436)

To determine if the hydrophobic residues of AH(427–436) are critical for SR-BI function, we mutated individual amino acids within the amphipathic helix to aspartic acid, a negatively charged, hydrophilic residue. These mutations would be expected to alter the intrinsic qualities of the amphipathic alpha helix, including mean hydrophobicity and mean hydrophobic moment. To quantify these changes, the sequences for AH(427–436) in WT-SR-BI or aspartic acid (D) mutants were submitted to the HeliQuest server (https://heliquest.ipmc.cnrs.fr/). Mean hydrophobicity and hydrophobic moment values increase with the number of hydrophobic residues and overall amphipathicity, respectively. For SR-BI aspartic acid mutants, the changes in mean hydrophobicity and hydrophobic moments, relative to WT-SR-BI, are listed in Table 1. As expected, hydrophilic residue mutants (blue) showed smaller reductions in mean hydrophobicity than in hydrophobic residue mutants (tan) (Table 1). Hydrophilic residue mutants led to slightly increased amphipathicity, while several hydrophobic residue mutants (L427D-SR-BI, F430D-SR-BI, Y431D-SR-BI, and L434D-SR-BI) led to decreased amphipathicity (Table 1). If L436 is indeed within the alpha helix and on the hydrophilic face of AH(427–436) (Fig. 1*B*), its mutation to aspartic acid would increase overall amphipathicity (Table 1). V435 also showed a slight increase in amphipathicity (Table 1), likely because this residue is closer to the hydrophilic face (Fig. 1*B*).

**Table 1:** Effects of aspartic acid mutations on mean hydrophobicity and amphipathicity of AH(427–436)

Helical face	Mutant	Change in mean hydrophobicity	Change in hydrophobic moment
Hydrophobic face	L427D	−0.247	−0.246
	F430D	−0.256	−0.030
	Y431D	−0.173	−0.096
	L434D	−0.247	−0.201
	V435D	−0.199	+0.033
Hydrophilic face	S428D	−0.073	+0.023
	T429D	−0.103	+0.098
	T432D	−0.103	+0.086
	Q433D	−0.055	+0.030
	L436D	−0.247	+0.247

Helical wheel analysis was performed using the HeliQuest server (https://heliquest.ipmc.cnrs.fr/) for residues 427 to 436 of WT-SR-BI, hydrophobic residue mutants, and hydrophilic residue mutants. The changes in mean hydrophobicity and hydrophobic moment values for each of the mutants as compared to WT-SR-BI are listed in the table. Rows of hydrophobic residues are shaded in *tan*, and hydrophilic residues are shaded in *blue*.

SR-BI, scavenger receptor class B type I.

### SR-BI aspartic acid mutants express in whole-cell lysates

**Figure 3:**
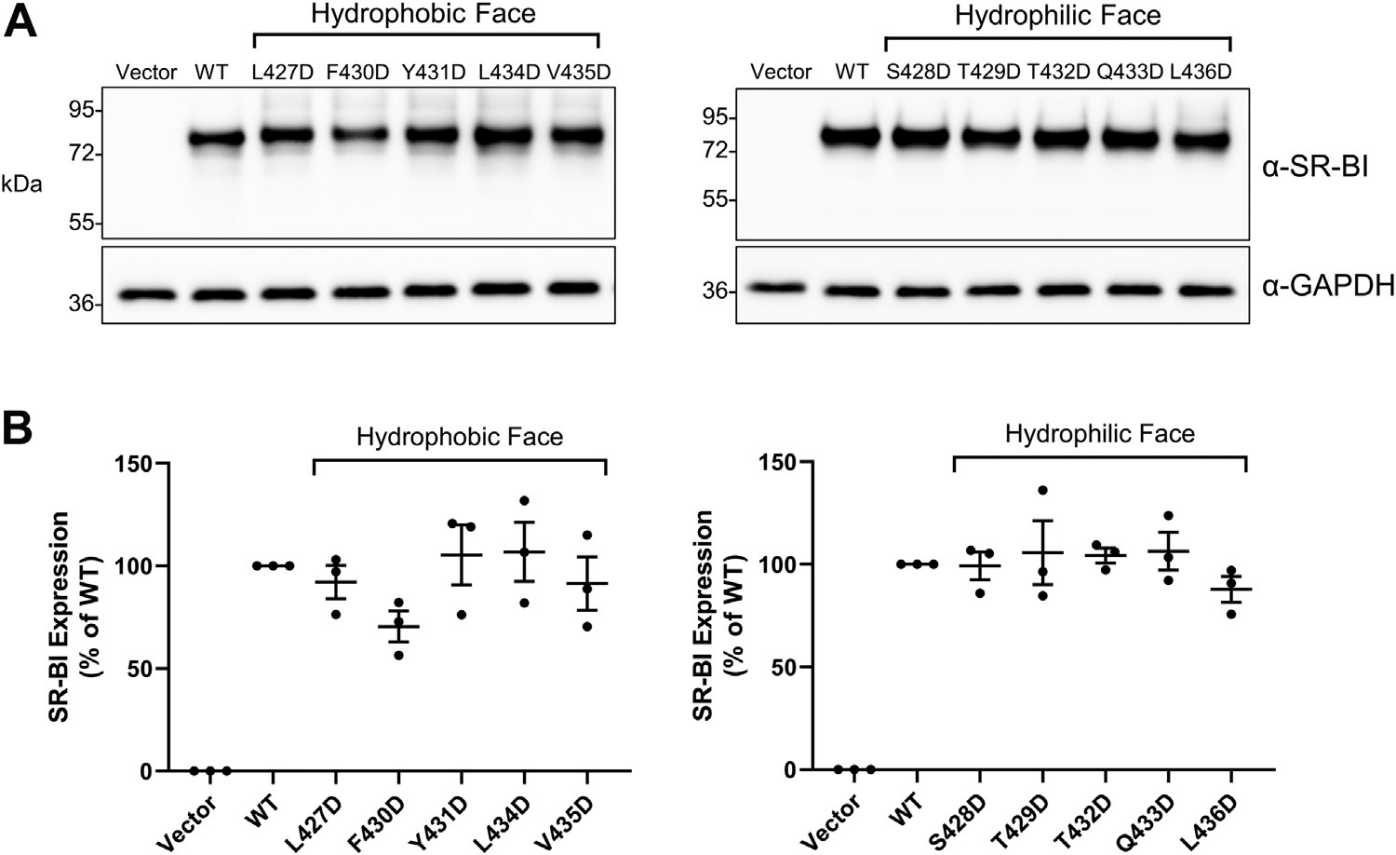
**SR-BI aspartic acid mutants express in whole-cell lysates**. *A*, immunoblot analysis using a C-terminal SR-BI targeting antibody was performed on whole-cell cleared lysates (10 μg) from COS-7 cells transiently expressing empty vector, WT-SR-BI, or individual aspartic acid mutants. GAPDH expression was probed as a loading control. Each blot is representative of three independent transfections (n = 3). *B*, densitometry of immunoblots was performed using NIH ImageJ software, and quantification of SR-BI expression relative to loading control is shown (WT-SR-BI = 100%). SR-BI, scavenger receptor class B type I; WT, wildtype.

Before assessing how loss of hydrophobic residues in AH(427–436) impacts SR-BI function, we first transiently transfected COS-7 cells with empty plasmid vector (pSG5) or plasmids encoding murine WT-SR-BI or mutant SR-BI to assess their total protein expression levels. After 48 h, cleared whole-cell lysates were collected and subjected to immunoblotting using a C-terminal targeting SR-BI antibody (residues 450–509), whose recognized epitope does not overlap with the mutated areas. Total expression levels of SR-BI mutants (Fig. 3*A*) were not statistically different than WT-SR-BI levels (Fig. 3*B*). However, F430D-SR-BI and L436D-SR-BI trended toward slight decreases in expression, relative to WT-SR-BI (mean ± SD = 70% ± 13% for F430D-SR-BI and 88% ± 11% for L436D-SR-BI).

### SR-BI aspartic acid mutants express at the cell surface

**Figure 4:**
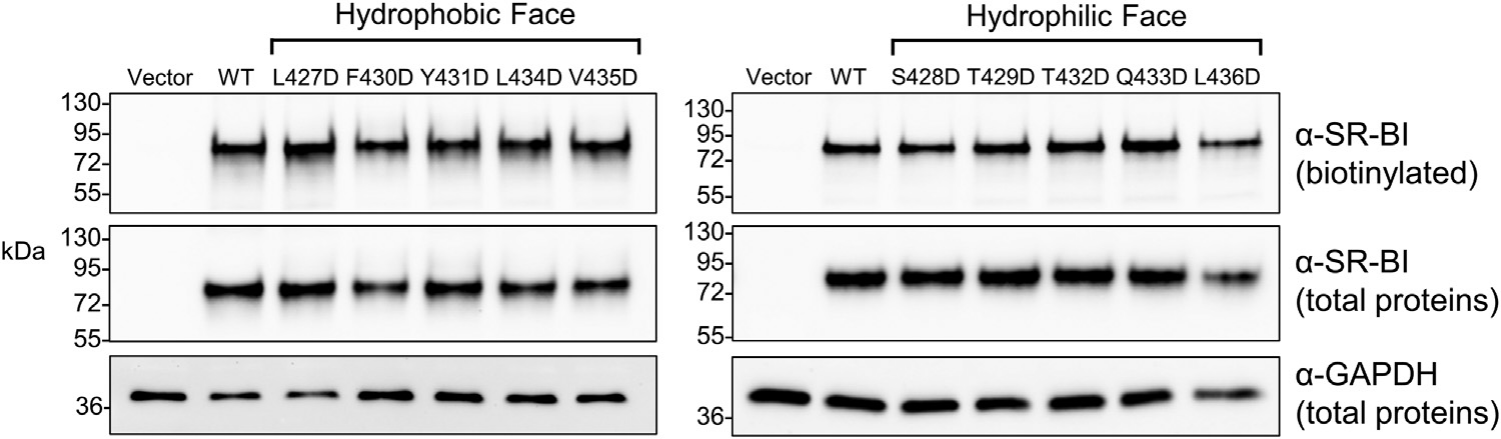
**SR-BI aspartic acid mutants express at the cell surface**. Cell surface biotinylation was performed on COS-7 cells transiently expressing empty vector, WT, or mutant SR-BI receptors. Biotinylated proteins (10 μl lysates, protein concentration [mean ± SD] = 9.4 μg ± 2.5 μg) were immunoblotted for SR-BI expression using a C-terminal SR-BI targeting antibody. A 10-μL aliquot of each total lysate, prior to biotin pull-down, was also immunoblotted for SR-BI (see “total proteins”, to account for differences in overall SR-BI expression) and GAPDH (to account for differences in the protein amounts incubated with streptavidin beads). Each blot is representative of three independent transfections (n = 3). SR-BI, scavenger receptor class B type I; WT, wildtype.

To specifically measure cell surface SR-BI expression, cell surface proteins were biotinylated with non-membrane-permeable EZ-Link Sulfo-NHS-LC-Biotin. Biotinylated proteins were pulled down in cell lysates by streptavidin-coated beads, eluted in Laemmli buffer, and separated by SDS-PAGE. Immunoblot analysis using the C-terminal targeting SR-BI antibody showed no major differences in cell surface expression between WT-SR-BI and the various mutant receptors (Fig. 4).

### SR-BI aspartic acid mutants retain the ability to dimerize or oligomerize

**Figure 5:**
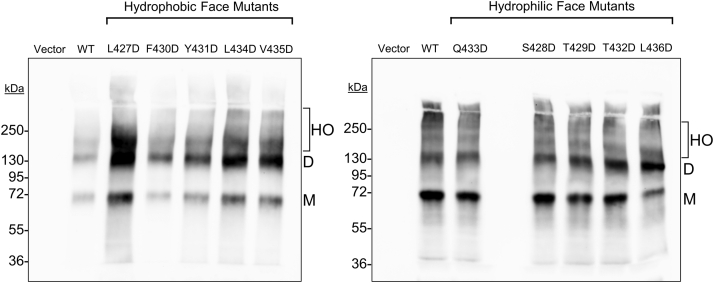
**SR-BI aspartic acid mutants retain the ability to dimerize or oligomerize**. COS-7 cells transiently expressing empty vector, WT, or aspartic acid SR-BI mutants were harvested in ice-cold PBS and lysed by sonication. Lysates (5 or 10 μg) were incubated in sample buffer containing PFOA (final concentration = 2.5%) and separated on 8% PFO-PAGE gels. Immunoblotting analysis using the C-terminal SR-BI targeting antibody was performed to assess the oligomerization profiles of SR-BI. Blots are each representative of three independent transfections (n = 3). SR-BI monomers (M), dimers (D), and higher-order oligomers (HO) are indicated. Note that hydrophilic mutants are not in numerical order. PFOA, perfluorooctanoic acid; SR-BI, scavenger receptor class B type I; WT, wildtype.

SR-BI is known to form dimers and higher-order oligomers, and several reports suggest that these may contribute to cholesterol transport (8, 15). In the cryo-EM structure of ATP-binding cassette transporter A1, two extracellular domains pack together forming a hydrophobic channel that may transport phospholipids and cholesterol (16). We envision that a similar phenomenon might occur with SR-BI. Within SR-BI’s AH(427–436), residues L427 and L434 are part of a leucine zipper dimerization motif that extends into the C-terminal transmembrane domain. Mutation of all leucine zipper motif residues was shown to disrupt SR-BI dimers and higher-order oligomers (8). More recently, large multimers averaging at least ten SR-BI molecules were reported at the plasma membrane of H295R adrenocortical cells (17). Upon mutation of the three C-terminal transmembrane leucine zipper residues (L441/L448/L455), the average size of the SR-BI multimers was reduced (17). The potential contribution of extracellular leucine zipper residues L427 and L434 to SR-BI dimerization has not been reported, and it is unknown if AH(427–436) contributes to SR-BI dimerization. Therefore, we sought to determine if mutation of residues within AH(427–436) to aspartic acid would disrupt SR-BI dimers and higher-order oligomers. To assess this, we performed PFO-PAGE analysis on lysates from COS-7 cells transiently expressing empty vector, WT-SR-BI, or aspartic acid mutants. The formation of dimers and higher-order oligomers was observed for all aspartic acid mutants (Fig. 5), demonstrating that individual residues in SR-BI-AH, including the leucine zipper motif residues, are dispensable for dimer formation. Although L427D-SR-BI expression appeared higher in some PFO-PAGE immunoblots, this was not always reproducible, and higher expression was not observed in whole-cell lysates (Fig. 3*A*).

### Mutation of key hydrophobic residues within AH(427–436) dramatically reduces HDL cell association and HDL-CE uptake

**Figure 6:**
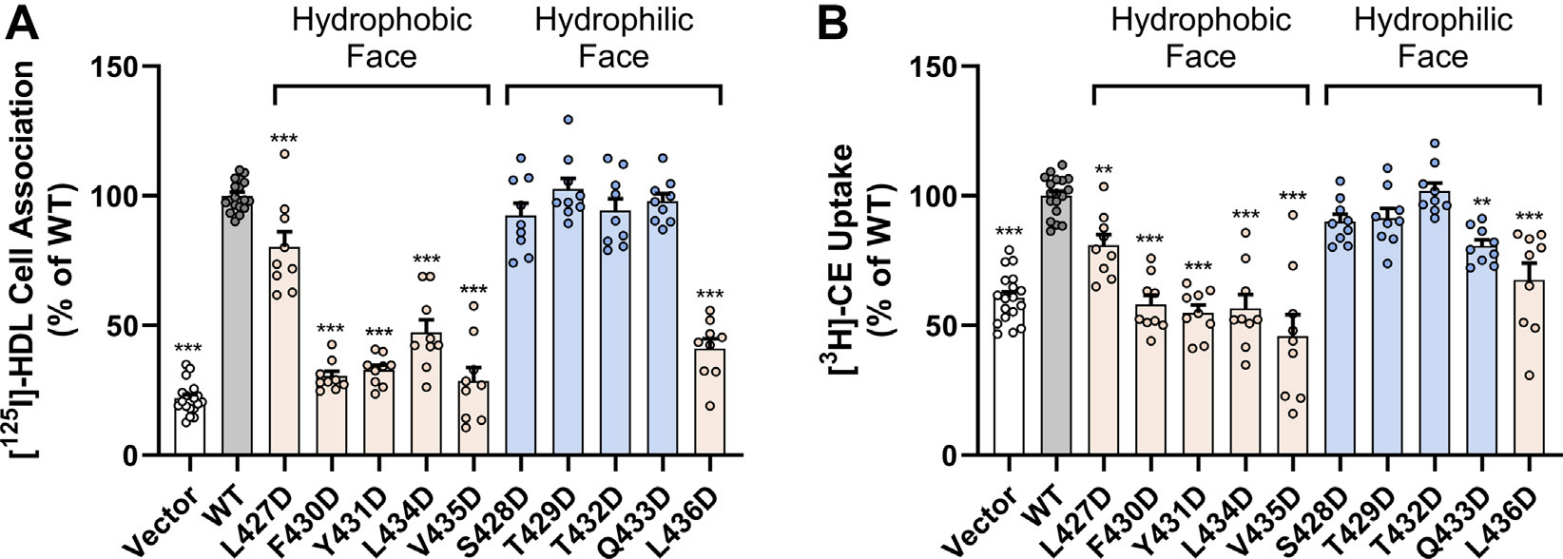
**Mutation of key hydrophobic residues within AH(427–436) dramatically reduces HDL cell association and HDL-CE uptake**. *A*, HDL cell association and *B*, CE uptake were measured in COS-7 cells expressing empty vector, WT-SR-BI, or aspartic acid mutants upon incubation with 10 μg/ml [^125^I]/[^3^H]-COE-HDL or [^125^I]/[^3^H]-CHE-HDL for 1.5 h at 37 °C. The specific activities (disintegrations per min/mg protein, mean ± SD) of two double-radiolabeled HDL preparations were [^125^I] = 97.0 ± 62.5 and [^3^H] = 257.9 ± 150.6. Radioactivity measurements were normalized to average WT-SR-BI values (WT-SR-BI = 100%) for HDL cell association and CE uptake. Data represent the mean ± SD for three independent transfections (n = 3), each performed in triplicate. As determined by one-way ANOVA with Dunnett’s multiple comparisons tests, ∗∗∗*p* < 0.001 and ∗∗*p* < 0.01 *versus* WT-SR-BI. CE, cholesteryl ester; CHE, cholesteryl hexadecyl ether; COE, cholesteryl oleyl ether; HDL, high-density lipoprotein; SR-BI, scavenger receptor class B type I; WT, wildtype.

To determine if the hydrophobic residues of AH(427–436) are important for SR-BI’s cholesterol transport functions, we first measured HDL cell association and HDL-CE uptake in COS-7 cells transiently expressing empty vector, WT-SR-BI, or aspartic acid mutants upon incubation with 10 μg/ml [^125^I]/[^3^H]-cholesteryl oleyl ether-HDL or [^125^I]/[^3^H]-cholesteryl hexadecyl ether-HDL for 1.5 h at 37 °C. Mutation of four residues on the hydrophobic face of AH(427–436) (F430, Y431, L434, and V435) and the lone hydrophobic residue on the hydrophilic face (L436) decreased mean HDL cell association to less than 50% of WT-SR-BI levels (Fig. 6*A*). There was only a slight but statistically significant decrease in HDL cell association for L427D-SR-BI, and no statistically significant difference was observed with mutation of hydrophilic residues on the hydrophilic face of AH(427–436) (Fig. 6*A*). Mutation of any hydrophobic residue in AH(427–436) led to statistically significantly reduced mean HDL-CE uptake (Fig. 6*B*). Strikingly, F430D-SR-BI, Y431D-SR-BI, L434D-SR-BI, and V435D-SR-BI demonstrated mean HDL-CE uptake levels that were below that of empty vector-transfected cells (Fig. 6*B*). One hydrophilic face mutant, Q433D-SR-BI, showed a slight decrease in HDL-CE uptake at about 80% of WT-SR-BI levels (Fig. 6*B*), despite this mutant demonstrating normal HDL cell association (Fig. 6*A*).

### SR-BI-mediated cholesterol efflux is impaired with aspartic acid mutations at L427, F430, Y431, and L434

**Figure 7:**
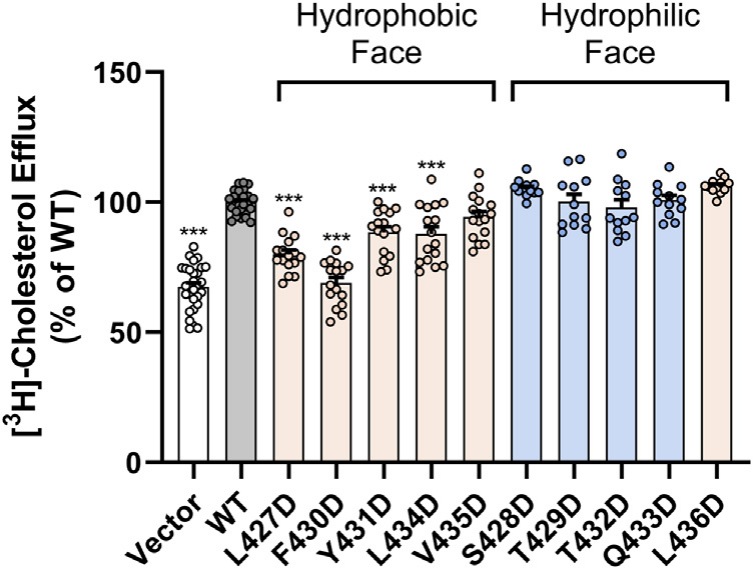
**SR-BI-mediated cholesterol efflux is impaired with aspartic acid mutations at L427, F430, Y431, and L434.** COS-7 cells expressing empty vector, WT-SR-BI, or aspartic acid mutants were pre-labeled with [^3^H]-cholesterol and incubated with HDL (50 μg/ml) for 4 h at 37 °C. Scintillation counts for the media and cellular lipids were used to calculate percent efflux (media counts/[cellular + media] counts x 100%). Percent efflux values were normalized to average WT-SR-BI values (WT-SR-BI = 100%). Data represent the mean ± SD for three to four independent transfections (n = 4), each performed in quadruplicate. As determined by one-way ANOVA with Dunnett’s multiple comparisons tests, ∗∗∗*p* < 0.001 *versus* WT-SR-BI. HDL, high-density lipoprotein; SR-BI, scavenger receptor class B type I; WT, wildtype.

To test the ability of aspartic acid mutants of SR-BI to mediate free cholesterol efflux to HDL, transiently transfected COS-7 cells were prelabeled with [^3^H]-cholesterol and incubated with human HDL (50 μg/ml) for 4 h at 37 °C. Percent free cholesterol efflux was statistically significantly decreased with L427D-SR-BI, F430D-SR-BI, Y431D-SR-BI, and L434D-SR-BI (Fig. 7). The effect was most dramatic with F430D-SR-BI, whose mean cholesterol efflux levels were similar to those of empty vector-transfected cells (Fig. 7). Mutation of residues on the hydrophilic face, including the hydrophobic residue (L436), had no statistically significant effect on cholesterol efflux (Fig. 7).

### Mutation of hydrophobic residues in AH(427–436) to aspartic acid impairs the ability of SR-BI to promote free cholesterol accessibility to cholesterol oxidase

**Figure 8:**
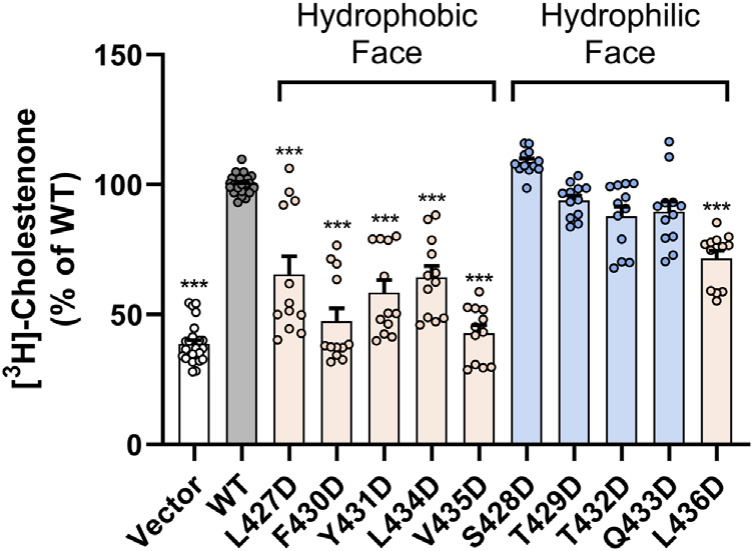
**Mutation of hydrophobic residues in AH(427–436) to aspartic acid impairs the ability of SR-BI to promote free cholesterol accessibility to cholesterol oxidase.** COS-7 cells expressing empty vector, WT-SR-BI, or aspartic acid mutants were prelabeled with [^3^H]-cholesterol and incubated with exogenous cholesterol oxidase (0.5 U/ml) for 4 h at 37 °C. Isopropanol-extracted cellular lipid species were separated by thin-layer chromatography analysis, and radioactivity of each species was measured by liquid scintillation counting. Cholestenone content was calculated as a percentage of total lipids. Average percent cholestenone values were normalized to average WT-SR-BI levels (WT-SR-BI = 100%). Data represent the mean ± SD of three independent transfections (n = 3), each performed in quadruplicate. As determined by one-way ANOVA with Dunnett’s multiple comparisons tests, ∗∗∗*p* < 0.001 *versus* WT-SR-BI. SR-BI, scavenger receptor class B type I; WT, wildtype.

SR-BI plays a role in enhancing the accessibility of free cholesterol within the plasma membrane to extracellular acceptors like HDL (18). To test the accessibility of membrane cholesterol using exogenous cholesterol oxidase, COS-7 cells expressing empty vector, WT-SR-BI, or aspartic acid mutants were prelabeled with [^3^H]-cholesterol and incubated with cholesterol oxidase (0.5 U/ml) for 4 h at 37 °C. Mutation of any AH(427–436) hydrophobic residue resulted in cholestenone production of approximately 70% or less of that in the presence of WT-SR-BI (Fig. 8), indicating reduced plasma membrane cholesterol accessibility. The most dramatic decreases were observed for F430D-SR-BI and V435D-SR-BI (Fig. 8). There were no statistically significant changes in cholestenone production for AH(427–436) hydrophilic residue mutants (Fig. 8).

## Discussion

The findings from our study suggest that certain hydrophobic residues within AH(427–436) are critically important for SR-BI-mediated HDL cholesterol transport functions. Based on homology modeling (Fig. 2*B*), a likely explanation is that some of these hydrophobic residues face the inner core of the SR-BI molecule, with F430 comprising part of a hydrophobic channel that unidirectionally or bidirectionally shuttles cholesterol between cells and HDL. Conversely, mutating the hydrophilic and uncharged residues on the opposite face had little to no impact on AH(427–436) amphipathicity or overall receptor function. Perhaps, this is because these residues are exposed in the aqueous extracellular space and, given their proximity to the plasma membrane, may not interact with HDL. Surprisingly, all hydrophobic mutants also showed a decrease in HDL cell association, despite being far from the theorized binding sites of HDL (3). Further investigation is needed to determine if there could be additional HDL binding sites or conformational changes that are disrupted by these mutations. Some mutations could have disrupted the alpha-helical structure of AH(427–436) or SR-BI folding, which could also affect its function.

Several mutants (F430D-SR-BI, Y431D-SR-BI, L434D-SR-BI, and V435D-SR-BI) exhibited CE uptake that was below the baseline of empty vector-transfected cells. F430D-SR-BI was the most impaired in cholesterol efflux, and F430D-SR-BI and V435D-SR-BI showed the strongest disruptions in membrane cholesterol accessibility (Figs. 7 and 8). Altogether, F430 emerged as a critical residue for SR-BI function, and our murine SR-BI homology model predicts that F430 is at the base of the hydrophobic tunnel (Fig. 2*B*). Previous studies where an overlapping region (F430-L434) was mutated to all alanine residues indicated that this region is important for CE uptake (19). Alanine substitutions in the adjacent region V435-Q439 also disrupted function, while alanine substitutions of K425-T429 had little impact on SR-BI function. This is consistent with our findings that L427D-SR-BI was less disruptive than other mutations and that S428D-SR-BI and T429D-SR-BI showed no statistically significant changes in function. Previously, our laboratory mutated leucine zipper motif residues L427 and L434 to alanine; neither mutation showed a statistically significant decrease in SR-BI function (8). In our current study, L427D-SR-BI showed intermediate functionality, while L434D-SR-BI showed only baseline levels of CE uptake. Thus, L427 appears to be more tolerant of severe mutations (*e.g.*, to aspartic acid) than L434. The fact that L427D-SR-BI function is not severely impaired, despite this mutation having the lowest mean hydrophobic moment (Table 1), seems to also indicate that overall amphipathicity of AH(427–436) is not critical for SR-BI function.

L436D-SR-BI was impaired in all functions except HDL cholesterol efflux, even though the NMR structure and helical wheel diagram place this residue on the hydrophilic face of AH(427–436). In the extracellular domain homology model, L436 is outside of the amphipathic helix in a flexible region next to the hydrophobic cavity (Fig. 2*B*). However, because L436 is near the end of the extracellular domain for which there are template X-ray crystal structures, there is a possibility of artifacts in the homology model. Thus, the homology model presented in Figure 2*A* may not accurately represent the secondary structure surrounding L436. If L436 is located on the hydrophilic face of the amphipathic helix, as the NMR structure suggests, then perhaps all hydrophobic residues in AH(427–436) are critical for function, regardless of the position. In a previous study, mutation of the nearby residue P438 to alanine resulted in impaired SR-BI-mediated cholesterol transport (20). Flexibility induced by P438 near the hydrophobic tunnel entrance at the plasma membrane could facilitate unidirectional or bidirectional cholesterol transport. Notably, Q433D-SR-BI showed normal HDL cell association but a reduction in HDL-CE uptake (Fig. 6). Although Q433 is on the hydrophilic face of AH(427–436), it is positioned toward the hydrophobic cavity opening (Fig. 2*A*), which could explain the slightly decreased CE uptake with Q433D-SR-BI. This, in addition to the fact that AH(427–436) hydrophobic moment did not correlate with function, indicates that there is no clear distinction of hydrophobic (and not hydrophilic) residues in AH(427–436) being associated with the hydrophobic cavity. While V435D-SR-BI showed decreased HDL cell association and CE uptake (Fig. 6, *A* and *B*), there was no significant difference in cholesterol efflux (Fig. 7). This is consistent with previous reports that HDL binding is required for CE uptake, but not cholesterol efflux (21, 22).

Previous studies of chimeric receptors suggested possible functional importance of a region in SR-BI that overlaps with AH(427–436). Chimeric SR-BI/CD36 receptors have been extensively studied because CD36 binds HDL but, unlike SR-BI, does not readily uptake HDL-CE (23, 24). Chimeric SR-BI/CD36 receptors were generated consisting of subdomains within SR-BI that were swapped with the corresponding sequence in CD36 (25). One chimeric receptor, harboring the CD36 sequence at residues 423 to 434 (that overlaps with eight residues of AH(427–436)), demonstrated impaired HDL cell association, CE uptake, and cholesterol efflux (25). While both the SR-BI and CD36 subdomain (423–434) sequences were predicted to be alpha-helical (25), analysis of their alpha-helical properties using the HeliQuest server (26) showed that the CD36 subdomain has low overall mean hydrophobicity (−0.027 vs. 0.512 for SR-BI) and a lower hydrophobic moment than SR-BI (0.338 vs. 0.491 for SR-BI). For AH(427–436), the mean hydrophobicity is 0.933 (vs. 0.294 for CD36), and the mean hydrophobic moment is 0.311 (vs. 0.329 for CD36). For this region, CD36 appears to have less overall hydrophobicity than SR-BI, which could potentially explain some differences between CD36 and SR-BI function.

It was previously hypothesized that AH(427–436) (referred to as “Helix 2” in the study by Chadwick *et al*. (8)) was a juxtamembrane domain (*i.e.*, a protein domain that directly interacts with the plasma membrane) (8). Evidence for this hypothesis arose from NMR experiments using a murine SR-BI peptide (residues 405–475), showing that AH(427–436) is more solvent-protected than would be anticipated if it were extending into the extracellular space and that it has the potential to interact with a membrane-like environment (8). These studies also demonstrated that an extracellular region–only peptide, SR-BI(405–445), only properly folded in the presence of detergent, meaning that the peptide may require a hydrophobic environment to fold (8). Our current study suggests that, in the context of full-length SR-BI, the required hydrophobic environment is more likely to be the inner hydrophobic core of SR-BI, rather than the plasma membrane. However, we cannot exclude the possibility that AH(427–436) interacts with the plasma membrane, but this would likely require conformational changes in SR-BI that remain unexplored.

## Conclusion

By mutating individual residues in AH(427–436) to aspartic acid, we have demonstrated that hydrophobicity of AH(427–436) is important for SR-BI’s ability to mediate cholesterol transport functions. We present the possibility that AH(427–436) makes up a portion of a hydrophobic tunnel that transports cholesterol through an SR-BI monomer. Although we focus on the impact of SR-BI mutations on cholesterol transport through the SR-BI monomer, it is also possible that multiple SR-BI extracellular domains pack together, forming a hydrophobic channel. More research is needed to investigate this potential cholesterol transport pathway. Understanding the mechanisms of cholesterol transport *via* SR-BI could facilitate the discovery of new therapies for cardiovascular disease.

## Experimental procedures

### Materials

COS-7 cells were obtained from the American Type Culture Collection. Rabbit polyclonal antibody targeting SR-BI’s C-terminal region (NB400–101) was purchased from Novus Biologicals. Rabbit-anti-GAPDH (#2118) antibody was obtained from Cell Signaling Technology. Horseradish peroxidase (HRP)–conjugated donkey-anti-rabbit-IgG was purchased from GE Healthcare Life Sciences. Fluorescein isothiocyanate (FITC)–conjugated goat-anti-rabbit IgG secondary antibody (#554020) was from BD Biosciences. [^125^I]-sodium iodide, [^3^H]-cholesteryl hexadecyl ether, [^3^H]-cholesteryl oleyl ether, and [^3^H]-cholesterol were purchased from PerkinElmer. Acyl-CoA cholesterol acyltransferase inhibitor (Sandoz 58–035) was obtained from MilliporeSigma. Human HDL was obtained from Alfa Aesar. Recombinant cholesteryl ester transfer protein was from Roar Biomedical. Cholesterol oxidase from *Streptomyces* spp. and thin-layer chromatography standards (cholesterol, 4-cholesten-3-one, and cholesteryl oleate) were obtained from Sigma-Aldrich. FuGENE 6 transfection reagent was obtained from Promega. EZ-Link Sulfo-NHS-LC-Biotin was purchased from Thermo Fisher Scientific. All other reagents were of analytical grade.

### SR-BI expression vectors

Single-point mutations to aspartic acid were generated in the coding region of murine SR-BI, which was previously cloned into the pSG5 expression vector (Strategene, Inc) (23). Top Gene Technologies (Pointe-Claire) performed DNA cloning, site-directed mutagenesis, and sequencing to verify mutations.

### COS-7 cell culture and transfection

COS-7 cells were maintained in DMEM containing sodium pyruvate, penicillin, streptomycin, and FBS at 37 °C/5% CO_2_. For transient transfections, cells were plated on 10-cm cell culture dishes and grown to approximately 70% confluency. Then, cells were transiently transfected with 10 μg of empty plasmid vector or plasmids containing WT or mutant SR-BI using FuGENE 6 transfection agent (1:3 ratio plasmid DNA:FuGENE 6). Unless otherwise specified, experiments were performed at 48 h post transfection.

### Cell lysis

COS-7 cells expressing empty vector, WT, or mutant SR-BI were washed twice in cold PBS and lysed in RIPA buffer with protease inhibitors for 10 min on ice. Cell lysates were cleared of cellular debris by centrifuging at 6010*g* for 10 min at 4 °C. Protein concentrations were determined by the Lowry method (27).

### SR-BI immunoblot analysis

Proteins were separated by 10% SDS-PAGE and wet-transferred onto nitrocellulose membranes. Antibody incubations were performed at the following concentrations: anti-C-terminal region of SR-BI (1:5000), anti-GAPDH (1:5000), and anti-rabbit-IgG-HRP (1:10000). Bands were visualized using enhanced chemiluminescence substrate on a ChemiDoc system (BioRad) or film developer and were quantified with NIH ImageJ software.

### Cell surface protein biotinylation

Cells expressing empty vector, WT, or mutant SR-BI were incubated with 1 mg/ml EZ-Link Sulfo-NHS-LC-Biotin (a non-membrane-permeable biotinylation reagent) for 1 h at 4 °C. Biotinylated cell surface proteins in 100 μl of cell lysate were pulled down by streptavidin beads, eluted by 2× Laemmli buffer with 10% β-mercaptoethanol, separated by SDS-PAGE, and probed with the C-terminal SR-BI antibody, as previously described (28).

### HDL cell association and HDL-CE uptake

Human HDL was double radiolabeled with [^3^H]-cholesteryl hexadecyl ether or [^3^H]-cholesteryl oleyl ether and [^125^I]-dilactitol tyramine, using established protocols (29, 30). The initial specific activities in disintegrations per min/mg protein (mean ± SD) were [^3^H] = 257.9 ± 150.6 and [^125^I] = 97.0 ± 62.5. HDL cell association and CE uptake in cells expressing WT or mutant SR-BI receptors were measured at 37 °C for 1.5 h, as previously described (23).

### Free cholesterol efflux to HDL

Cells expressing WT or mutant SR-BI receptors were labeled with [^3^H]-cholesterol. At 72 h post transfection, the cells were incubated with unlabeled human HDL (50 μg/ml) for 4 h at 37 °C to measure free cholesterol efflux, as previously described (20).

### Membrane cholesterol accessibility to cholesterol oxidase

Cells expressing WT or mutant SR-BI receptors were labeled with [^3^H]-cholesterol and incubated with cholesterol oxidase (0.5 U/ml) for 4 h at 37 °C. Resulting lipid extracts were separated by thin-layer chromatography, according to published protocols (20).

### Detecting SR-BI dimers and oligomers with perfluorooctanoic acid

Cells expressing WT or mutant SR-BI receptors were resuspended in PBS and sonicated for four cycles (3 s per cycle). Resulting cell lysates (10 μg) were mixed with equal volumes of perfluorooctanoic acid–containing buffer (5% perfluorooctanoic acid, 100 mM Tris base, 20% glycerol, 0.005% bromophenol blue), separated by PFO-PAGE (20, 31), and detected with the C-terminal SR-BI antibody.

### Data normalization and statistical analyses

Raw data values were normalized to WT levels of activity (WT-SR-BI = 100%). Statistical analysis was performed by one-way ANOVA and Dunnett’s multiple comparisons tests. Normalized data values are shown as the mean ± SD, where ∗*p* < 0.05, ∗∗*p* < 0.01, and ∗∗∗*p* < 0.001 *versus* WT-SR-BI.

## Data availability

All data are contained within the manuscript.

## Conflicts of interest

The authors declare that they have no conflicts of interest with the contents of this article.
